# A bioengineering method for modeling alveolar Rhabdomyosarcoma and assessing chemotherapy responses

**DOI:** 10.1016/j.mex.2021.101473

**Published:** 2021-07-27

**Authors:** Evan Stefanek, Ehsan Samiei, Mahboubeh Kavoosi, Mohammad Esmaeillou, Kiarash Roustai Geraylow, Arya Emami, Milad Ashrafizadeh, David Perrin, Joseph W Gordon, Mohsen Akbari, Saeid Ghavami

**Affiliations:** a Laboratory for Innovations in Micro Engineering (LiME), Department of Mechanical Engineering, University of Victoria, Victoria, Canada; b Centre for Advanced Materials and Related Technologies (CAMTEC), University of Victoria, Victoria, BC V8P 5C2, Canada; c Department of Biology, School of Basic Sciences, Research and Science Branch of Islamic Azad University, Zanjan, Iran; d Department of Medical Biotechnologies, University of Siena, Siena, Italy; e Student Research Committee, Semnan University of Medical Sciences, Semnan, Iran; f Faculty of Psychology, Department of Health, York University, ON, Canada; g Department of Human Anatomy and Cell Science, Max Rady College of Medicine, Rady Faculty of Health Sciences, University of Manitoba, Winnipeg, Manitoba, Canada; h Faculty of Engineering and Natural Sciences, Sabanci University, Orta Mahalle, Üniversite Caddesi No. 27, Orhanlı, Tuzla, Istanbul 34956, Turkey; i Department of Surgery, Section of Orthopaedic Surgery, University of Manitoba, Winnipeg MB R3A 1R9, Canada; j The Diabetes Research Envisioned and Accomplished in Manitoba (DREAM) Theme of the Children's Hospital Research Institute of Manitoba, Canada; k Biotechnology Center, Silesian University of Technology, Akademicka 2A, Gliwice 44-100, Poland; l Research Institute of Oncology and Hematology, Cancer Care Manitoba, University of Manitoba, Winnipeg MBR3E 0V9, Canada; m Autophagy Research Center, Shiraz University of Medical Sciences, Shiraz 7134845794, Iran; n Faculty of Medicine, Katowice School of Technology, Katowice, Poland

**Keywords:** Biofabrication, Rhabdomyosarcoma, Apoptosis, Autophagy, Cell death, AKT, Protein Kinase B, BSA, Bovine serum albumin, DAPI, 4’,6-Diami- dino-2-Phenylindole, Dihydrochloride, dECM, Decellularized extracellular matrix, DNA, Deoxyribonucleic acid, DFS, Disease-free survival, DMEM, Dulbecco's phosphate buffered saline, EM, Engineered model, EthD-1, Ethidium homodimer-1, EDTA, Ethylenediaminetetraacetic acid, ECM, Extracellular matrix, FBS, Fetal bovine serum, FOXO1, Forkhead box protein O1, HEPES, (4-(2-hydroxyethyl)-1-piperazineethanesulfonic acid), IgG, Immunoglobulin G, ICC, Immunocytochemistry, LC3, Microtubule associated protein 1A/1B-light chain 3, MEK, Mitogen-activated extracellular signal-regulated kinase, MYOD1, Myogenic muscle differentiation transcription factor 1, PAX, Paired box gene, PDMS, Polydimethylsiloxane, PNIPAAm, Poly-N-isopropylacrylamide, RGD, Arginylglycylaspartic acid, RMS, Rhabdomyosarcoma, RT, Room temperature, RPMI, Roswell Park Memorial Institute, TMZ, Temozolomide, 3D, Three-dimensional, 2D, Two-dimensional

## Abstract

Rhabdomyosarcoma (RMS) is the most common pediatric soft-tissue malignant tumor. Treatment of RMS usually includes primary tumor resection along with systemic chemotherapy. Two-dimensional (2D) cell culture systems and animal models have been extensively used for investigating the potential efficacy of new RMS treatments. However, RMS cells behave differently in 2D culture than in vivo, which has recently inspired the adoption of three-dimensional (3D) culture environments. In the current paper, we will describe the detailed methodology we have developed for fabricating a 3D engineered model to study alveolar RMS (ARMS) in vitro. This model consists of a thermally cross-linked collagen disk laden with RMS cells that mimics the structural and bio-chemical aspects of the tumor extracellular matrix (ECM). This process is highly reproducible and produces a 3D engineered model that can be used to analyze the cytotoxicity and autophagy induction of drugs on ARMS cells. The most improtant bullet points are as following:

• We fabricated 3D model of ARMS.
• The current ARMS 3D model can be used for screening of chemotherapy drugs.
• We developed methods to detect apoptosis and autophagy in ARMS 3D model to detect the mechansims of chemotherapy agents.

## Graphical abstract

Rhabdomyosarcoma (RMS) is the most common pediatric soft-tissue malignant tumor. Despite numerous clinical trials that have investigated the efficacy of multidrug chemotherapy regimens, outcomes for high-risk RMS patients are still poor which has inspired research into new targeted therapies that molecularly target malignant RMS cells. In order to evaluate such therapies *in vitro* in a realistic tumor microenvironment, we have bioengineered a cell-laden 3D tissue-engineered model with uniform geometry and an uncomplicated and reproducible biofabrication process. The transparent collagen-I hydrogel promotes cell attachment and growth and allows for analysis of the cellular response to drugs through brightfield microscopy, immunocytochemistry, and live/dead viability assays.

**Figure:**
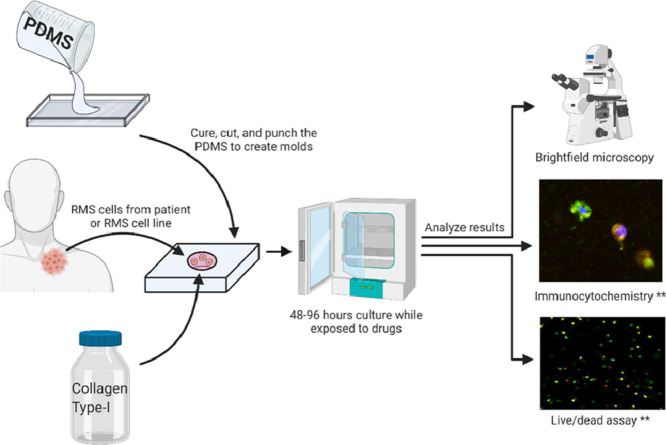

**Table:** Specifications Table

Subject area:	Pharmacology, Toxicology and Pharmaceutical Science
More specific subject area:	*Chemotherapy detection in 3D culture*
Protocol name:	Alveolar Rhabdomyosarcoma 3D model
Reagents/tools:	• Silicone elastomer kit, polydimethylsiloxane (PDMS) (SYLGARD™ 184, Dow Corning).• Vacuum chamber (Model 280 A, Thermo Fisher Scientific).• Glass slides (12-550C, Thermo Fisher Scientific).• Scalpel (08-927-5B, Thermo Fisher Scientific).• Neutralized Type I Collagen Solution, 5 mg/ml, Bovine (PureCol EZ Gel, Advanced Biomatrix).• 5 mm diameter biopsy punches (21909-142, VWR).Cell Culture:• Human alveolar Rhabdomyosarcoma cell line (RH30) [RC13, RMS 13, SJRH30] (ATCC® CRL- 2061™).• Mouse muscle cell line (C2C12) (ATCC® CRL-1772™).• Roswell Park Memorial Institute (RPMI-1640) with L-glutamine and 25 mM HEPES (12-115Q, BioWhittaker).• Dulbecco's Modified Eagle's Medium (DMEM, 50-003-PB, CORNING).• Penicillin-streptomycin (15140-122, Thermo Fisher Scientific).• Fetal bovine serum (FBS) (3160501, Thermo Fisher Scientific).• Trypsin-EDTA (15400-054, Thermo Fisher Scientific).• Incubator (Model #3403, Thermo Fisher Scientific).• T75 flasks (10062-160, VWR).
	Live/Dead Viability Assay:• Live/dead viability kit (L3224, Thermo Fisher Scientific).• Dulbecco's phosphate buffered saline (DPBS) (50-003-PB, CORNING).• Confocal microscope (LSM 980, Zeiss).
	Immunocytochemistry • IgG free bovine serum albumin (BSA) (Jackson ImmunoResearch Inc.). • Formaldehyde 37% (CAAAAA16163, VWR). • Triton X-100 (9002-93-1, Bio Basic Canada Inc.) • Primary antibodies (Cell Signalling Technology): • p62 (5114, 1:1000 dilution). • Cleaved PARP (Asp 214) (D64E10) XP® (5625, 1:100 dilution). • LC3B (D11) XP® (3868, 1:100 dilution). • Secondary antibodies (Jackson ImmunoResearch Inc.): • Alexa Fluor® 488 AffiniPure Donkey Anti-Rabbit IgG. • Alexa Fluor® 647 AffiniPure Donkey Anti-Mouse IgG. • 4’,6-Diami- dino-2-Phenylindole, Dihydrochloride (DAPI) (D1306, Thermo Fisher Scientific).
Experimental design:	The fabrication workflow for creating 3D TEM for studying RMS in vitro. (A) PDMS is poured onto a glass slide. The cured polymer is cut into sections which are each punched with a biopsy punch to create the molds. (**B**) Collagen hydrogel is mixed with cells and pipetted into each mold. The hydrogel filled molds are placed in 12 well plates and thermally crosslinked for 45 min at 37 °C before being submerged with cell media for culture. (**C**) After 12 h of culture in the 3D constructs, cytotoxic drugs are added as desired to the culture media. Following a 48 or 96 h exposure to the cytotoxic drugs, the cells are analyzed using brightfield microscopy, immunocytochemistry, or live/dead viability assays (Fig. 1).
Trial registration:	N/A
Ethics:	N/A
Value of the protocol:	The most important points about the protocol are as following: 1- Our method provides a simple and easy method for 3D culture of alveolar Rhabdomyosarcoma to detect chemotherapy response, 2- Our method established an easy way to measure autophagy and apoptosis in alveolar Rhabdomyosarcoma cells in 3D culture.

## Introduction

Rhabdomyosarcoma (RMS) is a rare and aggressive malignant soft tissue tumor that is usually diagnosed in children [1,2]. The combined annual incidence rate of all RMS cancers is 4.5 cases per million children [3,4]. Despite being the most common pediatric soft tissue tumor, the rarity of RMS increases the difficultly of recruiting patients for clinical studies and, in many cases, their results are limited by small samples sizes. The most common subtype is embryonal RMS which accounts for over 60% of all RMS diagnoses and has a 5-year survival rate of 73.4% [4,5]. This is a favorable diagnosis in comparison with alveolar RMS (ARMS), a less common pediatric subtype with significantly poorer outcomes and a 5 year survival rate of only 47.7% [4].

Typical treatment of childhood RMS is a multimodal therapy including chemotherapy and surgical tumor resection with or without radiation [6], [7], [8]. Patients with either ARMS or metastasis at diagnosis are considered to be at high risk with poor prognosis and therefore, warrants more intensive treatment [6,9]. Chemotherapy for pediatric RMS typically consists of an alkylating agent such as cyclophosphamide or ifosfamide combined with vincristine and actinomycin [6]. Alkylating agents act by covalently modifying DNA, which interferes with DNA replication and transcription [10]. This is particularly effective in quickly replicating tumor cells with p53 mutations or other damages to the DNA repair mechanisms, however this treatment can also induce side effects on other rapidly dividing non-cancerous cells such as hematopoietic progenitors or reproductive cells [10].

Clinical evidence suggests that there is significant potential to improve the medical treatment of RMS patients. Further exploration of innovative and targeted treatment techniques is necessary to meet this clinical need. Tissue engineering techniques are well poised to aid in the discovery and evaluation of new RMS treatments that will improve patient outcomes and survival rates

## Fabricating a 3D bioengineered *in vitro* model of RMS to detect programmed cell death

**Fig. 1:**
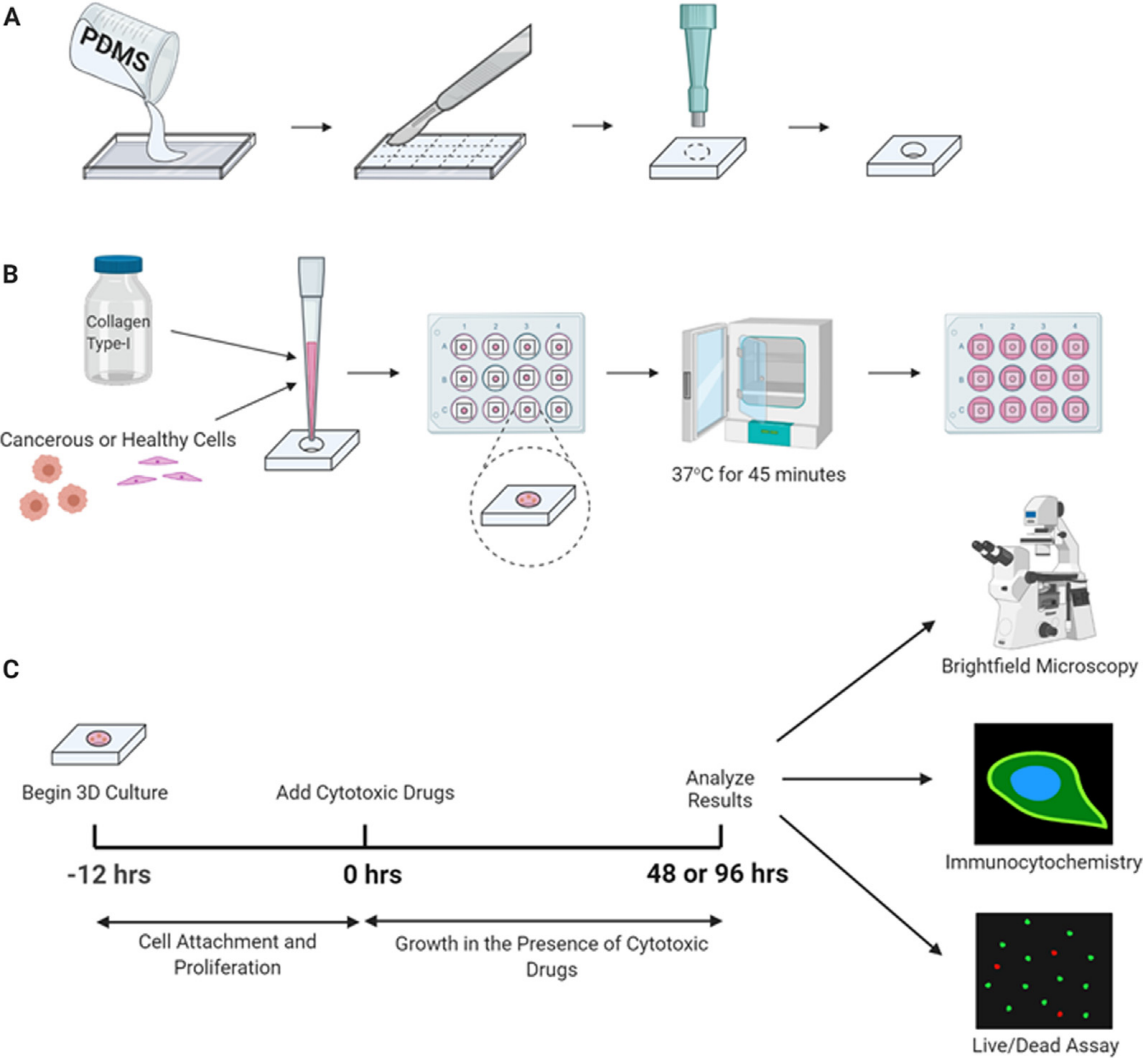
The fabrication workflow for creating 3D TEM for studying RMS in vitro. (A) PDMS is poured onto a glass slide. The cured polymer is cut into sections which are each punched with a biopsy punch to create the molds. (B) Collagen hydrogel is mixed with cells and pipetted into each mold. The hydrogel filled molds are placed in 12 well plates and thermally crosslinked for 45 min at 37 °C before being submerged with cell media for culture. (C) After 12 h of culture in the 3D constructs, cytotoxic drugs are added as desired to the culture media. Following a 48 or 96 h exposure to the cytotoxic drugs, the cells are analyzed using brightfield microscopy, immunocytochemistry, or live/dead viability assays.

We set out to design a 3D engineered model (EM) for studying ARMS *in vitro* that mimicked the *in vivo* tumor microenvironment and could be fabricated reproducibly through a robust process. The ECM of ARMS tumors *in vivo* is largely dependent on the ECM produced by the host's adjacent stromal cells, albeit slightly modulated by the secretions of cancerous cells [11]. Since collagen is a major component of the ECM of several human tissues, it is a great candidate for the formation of EM. In a report by Stracca-Pansa et al. [12], they showed that in 9 cases of ARMS there is only 1 patient with primarily Collagen V ECM and 1 patient with primarily laminin ECM. In another study, investigators showed that the ECM secretion by explant of pediatric tumors are different. They showed that the explant of different types of Rhabdomyosarcoma produced both interstitial collagen type I and III and basement membrane collagen type IV [13]. To identify the proper ECM to use in our ARMS 3D culture investigations, we performed experiments on human ARMS cell lines (RH30) and cultured them for 72 h and then collected their culture media and measure active transforming growth factor beta 1 (TGF-β1) as TGF-β1 is involved in biosynthesis of ECM [14], [15], [16]. Our results showed that RH30 cells secrets TGF-β1 (> 1900 pg/ml) and stimulates collagen type I precursor secretion in the media of RH30 cells (Ghavami and Gordon unpublished data). Therefore, it is reasonable to expect there is a significant quantity of collagen-I in the ECM of ARMS tumors. Collagen-I hydrogel was chosen as the biomaterial for our 3D TEM as it mimics the physiological ECM of the tumor site, contains Arginylglycylaspartic acid (RGD) peptides for *in vitro* cell attachment [17], and can form 3D geometries through thermal crosslinking. The most straightforward biofabrication technique would be to pipette the cell-laden hydrogel precursor directly into a 96-well plate prior to culture, as demonstrated by Musah-Eroje and Watson [18]. However, this would only allow nutrient and oxygen penetration from one direction and also would produce a non-uniform construct due to the hydrogel's meniscus and the geometry of the base of the well. In order to create a construct with symmetrical and uniform geometry, we designed polydimethylsiloxane (PDMS) molds that can be used to form disks of cross-linked cell-laden hydrogel with a consistent thickness and diameter (Fig. 1A,B). After fabrication, these 3D constructs can be cultured in a 12 well plate and media will evenly penetrate the uniform 3D geometry from the upper and lower surfaces of the disk (Fig. 1B). The transparent collagen hydrogel allows for detailed analysis of the suspended cells through brightfield microscopy, immunocytochemistry (ICC), and live/dead viability assays (Fig. 1C). A detailed description of the fabrication process along with the equipment and reagents required is presented below.

### Materials and equipment

#### 3D tissue engineered model

• Silicone elastomer kit, polydimethylsiloxane (PDMS) (SYLGARD™ 184, Dow Corning).
• Vacuum chamber (Model 280 A, Thermo Fisher Scientific).
• Glass slides (12-550C, Thermo Fisher Scientific).
• Scalpel (08-927-5B, Thermo Fisher Scientific).
• Neutralized Type I Collagen Solution, 5 mg/ml, Bovine (PureCol EZ Gel, Advanced Biomatrix).
• 5 mm diameter biopsy punches (21909-142, VWR).

Cell Culture:

• Human alveolar Rhabdomyosarcoma cell line (RH30) [RC13, RMS 13, SJRH30] (ATCC® CRL- 2061™).
• Roswell Park Memorial Institute (RPMI-1640) with L-glutamine and 25mM HEPES (12-115Q, BioWhittaker).
• Penicillin-streptomycin (15140-122, Thermo Fisher Scientific).
• Fetal bovine serum (FBS) (3160501, Thermo Fisher Scientific).
• Trypsin-EDTA (15400-054, Thermo Fisher Scientific).
• Incubator (Model #3403, Thermo Fisher Scientific).
• T75 flasks (10062-160, VWR).

Live/Dead Viability Assay:

• Live/dead viability kit (L3224, Thermo Fisher Scientific).
• Dulbecco's phosphate buffered saline (DPBS) (50-003-PB, CORNING).
• Confocal microscope (LSM 980, Zeiss).

Immunocytochemistry

• IgG free bovine serum albumin (BSA) (Jackson ImmunoResearch Inc.).
• Formaldehyde 37% (CAAAAA16163, VWR).
• Triton X-100 (9002-93-1, Bio Basic Canada Inc.)
• Primary antibodies (Cell Signalling Technology):
  ○ p62 (5114, 1:1000 dilution).
  ○ LC3B (D11) XP® (3868, 1:100 dilution).
• Secondary antibodies (Jackson ImmunoResearch Inc.):
  ○ Alexa Fluor® 488 AffiniPure Donkey Anti-Rabbit IgG.
  ○ Alexa Fluor® 647 AffiniPure Donkey Anti-Mouse IgG.
• 4’,6-Diami- dino-2-Phenylindole, Dihydrochloride (DAPI) (D1306, Thermo Fisher Scientific).
• LysoTracker Deep Red (Thermofisher Scientific, L12492)
• LC3-GFP (gift from Dr. Gordon's Lab)

#### Method

Fabrication of PDMS Molds

1. Combine a 10:1 ratio of the PDMS elastomer and curing agent as per the manufacturer's instructions, and mix well.
2. Degas the solution in a vacuum chamber until all bubbles are removed.
3. Pour the solution over a glass slide to create a 1 mm thick layer. Aluminum foil or PDMS can be used to form appropriately sized walls around the glass slide. Use the area of the glass slide to calculate the volume of solution required to get a thickness of 1 mm. For example, a 50 × 75 mm glass slide requires 3.75 ml of liquid PDMS.
4. Cure the elastomer mixture in an oven or over a hot-plate at 70 °C for 1,2 h.
5. With a scalpel, carefully cut the PDMS layer into 15 mm squares.
6. Punch a hole in the center of each PDMS square using a 5 mm biopsy punch.
7. To sterilize the mold, autoclave them at 120 °C using a steam autoclave.
  - If an autoclave is unavailable, incubate the PDMS molds with 70% ethanol for 1 h then rinse them with 100% ethanol and dry in an oven at 70 °C for at least 6 h. To avoid contamination, the molds should be kept in a sealed container.
8. Under aseptic conditions, transfer the molds to a 12-well plate.

2D Cell Culture

1. Working in aseptic conditions, prepare media: RPMI-1640 with 10% FBS, 1% penicillin/streptomycin.
2. In T-75 flasks, culture RH30 cells in complete RPMI-1640 and C2C12 cells in complete DMEM until they reach 80% confluency.
3. Remove media and wash the flask with 4 mL of PBS or trypsin-EDTA.
4. Incubate with 5 mL trypsin-EDTA for 5 min in an incubator, then pipette in the flask very gently to detach the cells.
5. Dilute the cell suspension with 10 mL media in a centrifuge tube.
6. Centrifuge at 200 g for 5 min.
7. Discard the supernatant.
8. The cells are ready for 3D culture or can be passaged for further 2D culture.

3D Cell Culture

**Note 1:** Collagen and all solutions that will be mixed with it should be maintained at 4 °C until crosslinking is desired.

1. Working in aseptic conditions, resuspend RH30 and C2C12 cells in 1 ml each of their respective media that is cooled to 4 °C.
2. Dilute the collagen solution with the cell-suspended media and additional media as required to produce final concentrations of 2 × 10^6^ cells/ml and 3 mg/ml collagen.

**Note 2:** The initial concentration of PureCol EZ Gel is 5 mg/ml collagen, and the pH and ionic/salt content is already adjusted to the application levels.

**Note 3:** If acidic collagen solutions are to be used (e.g. FibriCol), prior to use the proper amounts of 10X PBS, NaOH and distilled water must be added to the collagen solution to adjust the salt/ionic concentration to the level of 1X PBS, and the pH to 7.4.

1. Add 20 μL of the cell-laden hydrogel into each PDMS mold.
2. Incubate in an incubator at 37 °C and 5% CO_2_ for 45 min to thermally crosslink the hydrogel.
3. Gently add 2 ml of the appropriate media to each well and culture for 12 h or overnight.
4. Add cytotoxic drugs or other reagents as desired.
5. Observe results at relevant timepoints using brightfield microscopy, live/dead viability assays, immunocytochemistry or other analysis techniques.

Live/Dead Viability Assay for 3D Culture

1. As per the manufacturer's instructions, prepare a solution of 2 μM calcein AM and 4 μM ethidium homodimer-1 (EthD-1) in DPBS. To produce 10 ml of staining solution, use 5 μl calcein AM and 20 μl Ethd-1.
2. Remove media from the wells and add the live/dead solution.
3. Incubate for 1,2 h in the dark at room temperature (RT).
4. Remove the live/dead solution and wash three times with DPBS for 5 min each.
5. Immediately image using a confocal microscope.

Immunocytochemistry

1. Remove media and fix cells with 3.7% formaldehyde in DPBS at RT for 40 min.
2. Remove formaldehyde solution and wash 3 times with DPBS for 5 min each.
3. Incubate with the blocking solution of 5% BSA and 0.3% Triton-X for 2 h at RT.
4. Dilute the primary antibody(s) as per the manufacturer's recommendation (1:300) in a DPBS solution containing 1% BSA and 0.3% Triton-X.

**Note:** Primary antibodies for p62 and LC3 should be combined in the same solution for co-staining while the PARP primary antibody should be diluted separately.

1. Incubate the samples overnight in their respective primary antibody solutions.
2. Remove the solution and wash 3 times with DPBS for 5 min each.
3. Dilute the secondary antibodies separately as per the manufacturer's recommendation (1:300) in a DPBS solution containing 1% BSA and 0.3% Triton-X.
4. Incubate the samples for 2–4 h in their respective secondary antibody solutions in the dark at RT.
5. Remove the secondary antibody solution and incubate with a DAPI solution for 1,2 h in the dark at RT.
6. Remove the DAPI solution and wash 3 times with DPBS for 5 min each.
7. Immediately image using a confocal microscope.

**Note:** The fixed cell-laden collagen disks can be cryo-sectioned after fixation for higher resolution imaging.

**Fig. 2:**
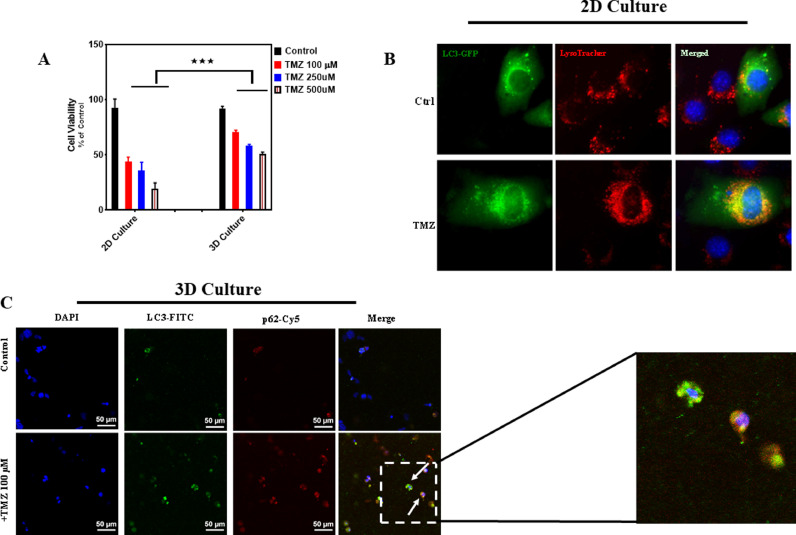
The effect of TMZ exposure on RH30 cell viability and autophagy in 2D and 3D culture . (A) RH30 viability in 2D and 3D culture after 96 h of exposure to TMZ concentrations (100, 250, 500 μM) was measured using live/dead assay. TMZ induced significant less cell death in 3D culture model (*** *p* < 0.0001). All experiments have been done in three independent biological replicates We used Two Way ANOVA test to compare viability between 2D and 3D culture model. (B, C) TMZ induced autophagy in both 2D (B) and 3D (C) RH30 cultures. RH30 cells were treated with TMZ (100 μM, 72 h) in 2D and 3D culture. We used immunocytochemistry and detected LC3 puncta and changes in lysosomal activity and intensity (LysoTracker red staining), co-localization of LC3 puncta and LysoTracker, and co-localization of LC3 and p62. These evidences showed that TMZ induced autophagy in 2D and 3D RH30 culture (19). (For interpretation of the references to color in this figure legend, the reader is referred to the web version of this article.)

We then demonstrated that alveolar RMS cells (RH30) in the developed 3D EM behaved differentially as in 2D cell culture in response to chemotherapy agent (TMZ) [19] (Fig. 2A). Our results showed that TMZ induced significantly higher cell death in 2D culture that 3D EM (p , 0.001). As the 3D EM closer mimics physiological conditions, it is expected that its results will be more representative of *in vivo* behavior. Firstly, we evaluated the effects of TMZ on RH30 2D and these results were compared with parallel experiments conducted in the 3D EM [19]. As explained in further details in the method above, to fabricate the 3D culture model RH30 cells were suspended in separate collagen-I hydrogels. The cell-laden hydrogels were deposited in polydimethylsiloxane (PDMS) molds and thermally cross-linked to form 3D constructs 5 mm in diameter and 1 mm in height. These constructs were submerged in cell media in a 12-well plate for culture and later evaluations of drug toxicity. Live/dead viability assays were used to quantify cell viability within the 2D and 3D culture environments respectively (Fig. 2A). When grown in 2D conditions, RH30 cells showed a significant decrease in viability upon exposure to TMZ while 3D culture showed significantly lower toxicity to TMZ treatment (*p* > 0.001) (Fig. 2A).

As the response of the cells is affected by macroautophagy (hereafter autophagy), we also evaluated autophagy in both 2D and 3D culture. Our results showed that TMZ induced autophagy in both 2D (Fig. 2B, induction of LC3 puncta and co-localization with lysotracker red) and 3D (Fig. 2C, LC3 puncta and co-localization with p62). We later counted the number of LC3 puncta in 10 different fields in both 2D and 3D conditions to compare autophagy between 2D and 3D culture. Interestingly, there was not any significant difference between 2D and 3D culture (*p* > 0.05) [19]. Therefore, the difference between the response to TMZ in 2D and 3D culture was not depended on the autophagy.

The proposed 3D EM has a relatively simple fabrication method in comparison with existing 3D culture models, which require delicately arranging cell sheets [20], extrusion bioprinting [21], or decellularizing tissues [22]. Furthermore, the proposed EM's uniform geometry allows for even nutrient and media penetration and simplifies imaging in comparison to non-uniform constructs formed by pipetting hydrogels directly into well-plates [18]. The fabrication process of the proposed EM is highly reproducible which allows for direct comparisons if used in different studies. Despite the ease of fabrication, the collagen-I hydrogel still mimics the tumor ECM to a similar degree as existing studies which have used alginate/gelatin/fibrinogen composite hydrogels [21] or murine basement membrane extract [18].

## Existing methods for studying RMS and other cancers

Poor outcomes for high-risk RMS patients have motivated researchers into finding potential treatments that are superior to the existing chemotherapy regimens. Two-dimensional (2D) cell culture of either immortalized RMS cell lines or patient-derived RMS tumors cells has been the most common strategy for investigating the efficacy of potential RMS treatments *in vitro* [23], [24], [25], [26], [27], [28], [29]. Generally, the workflow of these studies begins by identifying a suitable pathway to target based on its importance in other cancers or through the genetic analysis of RMS patient tumors. This is followed by selecting or developing a chemical agent that targets this pathway and observing its effect on the survival and phenotype of RMS cells in 2D cell culture. This framework was followed in a study of murine double minute 2 (MDM2) inhibitors that first identified a common genetic mutation in RMS patients whose gene product interacts with MDM2 [23]. An MDM2 inhibitor was added to 2D cultures of various immortalized RMS cell lines which was shown to cause a significant decrease in cell proliferation. Another study hypothesized that histone deacetylase inhibitors, which are efficacious at treating other adult solid tumors, may also be a useful for treatment of RMS [26]. 2D culture of immortalized RMS cell lines was used to evaluate the effects of a histone deacetylase inhibitor *in vitro*. The results showed that this inhibitor induced cell death and reduced growth.

To more convincingly demonstrate the effects of potential RMS therapies, there has been substantial use of animal models, primarily immunodeficient mice, to show how RMS tumors grow and respond to treatments *in vivo*. Animal models of RMS have many advantages over 2D cell culture as they allow cancerous cells to grow in a three-dimensional (3D) tumor environment and mimic the heterogenous cell population at the tumor site. One such *in vivo* study first investigated in 2D culture how RMS cells derived from 20 patients responded to a database of over 200 drugs [27].

Despite the advantages of using animal models over 2D cell culture, biological distinctions between humans and animals limit the usefulness of results from *in vivo* studies. The ethical considerations of using numerous animals for research further diminishes the feasibility of continuing to use these models in the future to gain insights about RMS treatment in humans. 3D tissue engineered models (EMs) have the potential to address both of these issues as they can encapsulate solely human cells and largely avoid concerns of animal welfare. Beyond this, 3D EMs are more reproducible that animal models and offer flexibility in the cell types, biomaterials, and geometries used. Cancer associated cells such as fibroblasts and macrophages have prominent roles in the *in vivo* tumor microenvironment [30,31] and can be incorporated in 3D EMs alongside a combination of cancerous or healthy human cells. Existing biomaterial technologies allow 3D scaffolds to be fabricated from natural, synthetic, or composite polymers that can provide a wide range of mechanical or electrical properties, desirable biological interactions, and have the potential for chemical modification [32]. The use of molds or 3D bioprinting enables cell-laden biomaterials to be arranged and cross-linked in complex 3D geometries that can be designed with computer modeling software. These advantages have not gone unnoticed, particularly in the field of oncology research where many *in vitro* 3D EMs laden with cancer cells have been reported [18,21,33,34]. The few existing 3D models of RMS other than our previously described 3D model [19,35] will be discussed in detail, followed by a discussion of a few of the many 3D cell culture models that have been used to study glioma.

All 3D cell culture models have the common advantage over 2D culture of allowing cells to attach to the substrate, migrate, and proliferate in a three-dimensional manner. Notable differences between models arise when considering the range of biomaterials used and their respective properties. Natural hydrogels can mimic the biochemical properties of the native ECM and some provide integrin binding to RGD peptides *in vitro* [17,36]. A biomaterial with a small pore size will limit the diffusion of oxygen and nutrients through the construct, a phenomenon seen *in vivo* within RMS tumors by the presence of hypoxic regions [37]. The fabrication complexity of a 3D EM is also an important factor that can determine the feasibility of use. A time-consuming or expensive biofabrication process such as growing tumor xenografts *in vivo* [22] or manually layering cell-sheets [20] will discourage their widespread use. A biofabrication procedure such as extrusion 3D bioprinting that requires technical expertise, specialized equipment, and extensive optimization will likewise have a slow rate of adoption. Simpler biofabrication techniques that allow for high-throughput analysis of RMS cells in 3D are more appropriate. Additionally, less complicated biofabrication techniques often produce results that are more consistent and reproducible.

## Conclusion and future directions

The protocol presented above produces a 3D EM suitable for culturing ARMS or healthy cells in 3D and studying their response to potential ARMS treatments. In contrast to existing models, our 3D EM has a comparatively simple fabrication method, is reproducible, and has a uniform 3D structure. Our previous studies have verified that ARMS or healthy cells can be encapsulated within this 3D EM and that live/dead viability assays or ICC can be used to observe the magnitude and mechanism of cell death upon exposure to cytotoxic drugs. Although this protocol was optimized for the use of RH30 and C2C12 cells, future adaptations could include other ARMS cell lines, patient-derived ARMS tumor cells, or healthy human myoblast cells grown in the 3D EM. The use of human myoblasts would permit meaningful co-cultures with ARMS cells to investigate the interactions between healthy and cancerous cells in a realistic tumor microenvironment. Another incremental improvement to the 3D EM described in this work could include increasing the stiffness of the hydrogel to closer mimic that of the *in vivo* tumor ECM by adding hyaluronic acid, a naturally occurring glycosaminoglycan, or transglutaminase, an enzyme which can crosslink collagen fibers.

While our 3D EM has the potential to provide preliminary results about the efficacy of a potential ARMS treatment, a more complex model that better mimics the *in vivo* tumor environment may be better suited to justify a treatment's progression to clinical trials. For instance, 3D bioprinting can deposit cells throughout EMs in a predefined pattern with high spatial resolution. A 3D bioprinted construct can also include relevant geometries such as vasculature, which would allow for the delivery of drugs via perfusion, analogous to the systemic delivery method of most chemotherapy drugs. Although the fabrication complexity of 3D bioprinted models is substantial, such efforts may be necessary to further investigate treatment efficacies *in vitro* following the discovery of new promising treatments. Similarly, microfluidic systems offer the ability to perform high-throughput tests as well as targeted studies of invasion, migration, compartmentalized co-culture, and gradient delivered cytokines. In comparison with our EM, these benefits come with an increased fabrication complexity; however, if the study can provide meaningful results and a potentially positive impact on patient prognosis, then the additional effort in fabrication would be well justified.

Investigation of specific signaling pathways in human disease and modulation of the genes involved in regulation of specific pathways to improve the effects of chemotherapy agents are one of the major aims in biomedical sciences [38], [39], [40]. Recent investigations have used modified primary and cell lines in 3D culture model of different diseases for example kidney diseases and breast cancer [41,42]. They have used gene editing technologies including clustered regularly interspaced short palindromic repeats (CRISPR)/CRISPR-associated systems 9 (Cas9) and siRNA to silence different genes. Therefore, ARMS 3D culture will be a good model to investigate the genetic role of specific genes involved in this cancer after preparation of single clones of specific genes involved in this disease (over expression/knock out) in 2D cultures and then use these modified cells in a 3D ARMS cell culture model.

It has been reported that epigenetics is very important in different types of RMS (including ARMS) [43]. For example, in a recent investigation it has been shown that an unfolded protein response is endogenously upregulated in ARMS [44]. Therefore, using different inhibitors which targets this pathway including MKC886 and GSP-PERK inhibitor in 3D culture of ARMS could be a very strong application of our model in developing future therapeutic opportunities for this lethal incurable pediatric cancer.

** Images reproduced from:

Moghadam, A.R., et al., Autophagy modulates temozolomide-induced cell death in alveolar Rhabdomyosarcoma cells. Cell Death Discov, 2018. **4**: *p*. 52.

## CRediT authorship contribution statement

**Evan Stefanek:** Conceptualization, Formal analysis, Methodology, Writing – original draft. **Ehsan Samiei:** Data curation, Visualization. **Mahboubeh Kavoosi:** Software, Writing – original draft, Writing – review & editing. **Mohammad Esmaeillou:** Software, Writing – original draft, Writing – review & editing. **Kiarash Roustai Geraylow:** Software, Writing – original draft, Writing – review & editing. **Arya Emami:** Software, Writing – original draft, Writing – review & editing. **Milad Ashrafizadeh:** Software, Writing – original draft, Writing – review & editing. **David Perrin:** Supervision, Writing – original draft, Writing – review & editing. **Joseph W Gordon:** Supervision, Writing – original draft, Writing – review & editing, Funding acquisition. **Mohsen Akbari:** Funding acquisition, Project administration, Supervision, Writing – review & editing. **Saeid Ghavami:** Funding acquisition, Project administration, Supervision, Writing – review & editing.

## Conflicts of interest

The authors declare that they have no known competing financial interests or personal relationships that could have appeared to influence the work reported in this paper.

## Appendix Supplementary materials

Image, image 1

## References

[1] Pappo A.S., Shapiro D.N., Crist W.M., Maurer H.M. (1995). Biology and therapy of pediatric Rhabdomyosarcoma. J. Clin. Oncol..

[2] Xia S.J., Pressey J.G., Barr F.G. (2002). Molecular pathogenesis of Rhabdomyosarcoma. Cancer Biol. Ther..

[3] Ries L.A.G., Smith M.A., Gurney J.G., Linet M., Tamra T., Young J.L. (1999). Cancer Incidence and Survival Among Children and Adolescents: United States SEER Program 1975-1995.

[4] Ognjanovic S., Linabery A.M., Charbonneau B., Ross J.A. (2009). Trends in childhood Rhabdomyosarcoma incidence and survival in the United States, 1975-2005. Cancer.

[5] Ramadan F., Fahs A., Ghayad S.E., Saab R. (2020). Signaling pathways in Rhabdomyosarcoma invasion and metastasis. Cancer Metastasis Rev..

[6] Bisogno G., De Salvo G.L., Bergeron C., Melcón S.G., Merks J.H., Kelsey A. (2019). Vinorelbine and continuous low-dose cyclophosphamide as maintenance chemotherapy in patients with high-risk Rhabdomyosarcoma (RMS 2005): a multicentre, open-label, randomised, phase 3 trial. Lancet Oncol..

[7] Bisogno G., Jenney M., Bergeron C., Gallego Melcón S., Ferrari A., Oberlin O. (2018). Addition of dose-intensified doxorubicin to standard chemotherapy for Rhabdomyosarcoma (EpSSG RMS 2005): a multicentre, open-label, randomised controlled, phase 3 trial. Lancet Oncol..

[8] Ohi S. (2007). Characterization, anticancer drug susceptibility and atRA-induced growth inhibition of a novel cell line (HUMEMS) established from pleural effusion of alveolar Rhabdomyosarcoma of breast tissue. Hum Cell.

[9] Bisogno G., Jenney M., Bergeron C., Melcón S.G., Ferrari A., Oberlin O. (2018). Addition of dose-intensified doxorubicin to standard chemotherapy for Rhabdomyosarcoma (EpSSG RMS 2005): a multicentre, open-label, randomised controlled, phase 3 trial. Lancet Oncol..

[10] Furlanut M., Franceschi L. (2003). Pharmacology of ifosfamide. Oncology.

[11] De L Davies C., Berk D.A., Pluen A., Jain R.K. (2002). Comparison of IgG diffusion and extracellular matrix composition in Rhabdomyosarcomas grown in mice versus in vitro as spheroids reveals the role of host stromal cells. Br. J. Cancer.

[12] Stracca-Pansa V., Dickman P.S., Zamboni G., Bevilacqua P.A., Ninfo V. (1994). Extracellular matrix of small round cell tumors of childhood: an immunohistochemical study of 67 cases. Pediatr. Pathol..

[13] DeClerck Y.A., Bogenmann E., Jones P.A. (1985). Collagen synthesis by short-term explants of pediatric tumors. Cancer Res..

[14] Ghavami S., Yeganeh B., Zeki A.A., Shojaei S., Kenyon N.J., Ott S. (2018). Autophagy and the unfolded protein response promote profibrotic effects of TGF-beta1 in human lung fibroblasts. Am. J. Physiol. Lung Cell. Mol. Physiol..

[15] Schaafsma D., McNeill K.D., Mutawe M.M., Ghavami S., Unruh H., Jacques E. (2011). Simvastatin inhibits TGFbeta1-induced fibronectin in human airway fibroblasts. Respir. Res..

[16] Ghavami S., Cunnington R.H., Gupta S., Yeganeh B., Filomeno K.L., Freed D.H. (2015). Autophagy is a regulator of TGF-beta1-induced fibrogenesis in primary human atrial myofibroblasts. Cell Death Dis..

[17] Davidenko N., Schuster C.F., Bax D.V., Farndale R.W., Hamaia S., Best S.M. (2016). Evaluation of cell binding to collagen and gelatin: a study of the effect of 2D and 3D architecture and surface chemistry. J. Mater. Sci. Mater. Med..

[18] Musah-Eroje A., Watson S. (2019). A novel 3D *in vitro* model of glioblastoma reveals resistance to temozolomide which was potentiated by hypoxia. J. Neurooncol..

[19] Moghadam A.R., Da Silva Rosa S.C., Samiei E., Alizadeh J., Field J., Kawalec P. (2018). Autophagy modulates temozolomide-induced cell death in alveolar Rhabdomyosarcoma cells. Cell Death Discov..

[20] Li M., Nagamori E., Kino-Oka M. (2017). Disruption of myoblast alignment by highly motile Rhabdomyosarcoma cell in tissue structure. J. Biosci. Bioeng..

[21] Wang X., Dai X., Zhang X., Ma C., Li X., Xu T. (2019). 3D bioprinted glioma cell-laden scaffolds enriching glioma stem cells via epithelial-mesenchymal transition. J. Biomed. Mater. Res. A.

[22] Pozzobon M., Saggioro M., D'Agostino S., Bisogno G., Muraca M., Gamba P. (2018). Alveolar Rhabdomyosarcoma Decellularization. Methods Mol. Biol..

[23] Canner J.A., Sobo M., Ball S., Hutzen B., DeAngelis S., Willis W. (2009). MI-63: a novel small-molecule inhibitor targets MDM2 and induces apoptosis in embryonal and alveolar Rhabdomyosarcoma cells with wild-type p53. Br. J. Cancer.

[24] Loupe J.M., Miller P.J., Ruffin D.R., Stark M.W., Hollenbach A.D. (2015). Inhibiting phosphorylation of the oncogenic PAX3-FOXO1 reduces alveolar Rhabdomyosarcoma phenotypes identifying novel therapy options. Oncogenesis.

[25] Shapiro D.N., Sublett J.E., Li B., Downing J.R., Naeve C.W. (1993). Fusion of PAX3 to a member of the forkhead family of transcription factors in human alveolar Rhabdomyosarcoma. Cancer Res..

[26] Kutko M.C., Glick R.D., Butler L.M., Coffey D.C., Rifkind R.A., Marks P.A. (2003). Histone deacetylase inhibitors induce growth suppression and cell death in human Rhabdomyosarcoma in vitro. Clin. Cancer Res..

[27] Manzella G., Schreck L.D., Breunis W.B., Molenaar J., Merks H., Barr F.G. (2020). Phenotypic profiling with a living biobank of primary Rhabdomyosarcoma unravels disease heterogeneity and AKT sensitivity. Nat. Commun..

[28] Adamus A., Engel N., Seitz G. (2020). SGPL1321 mutation: one main trigger for invasiveness of pediatric alveolar Rhabdomyosarcoma. Cancer Gene Ther..

[29] Igarashi K., Kawaguchi K., Kiyuna T., Murakami T., Miwa S., Nelson S.D. (2017). Temozolomide combined with irinotecan caused regression in an adult pleomorphic Rhabdomyosarcoma patient-derived orthotopic xenograft (PDOX) nude-mouse model. Oncotarget.

[30] Galletti G., Scielzo C., Barbaglio F., Rodriguez T.V., Riba M., Lazarevic D. (2016). Targeting macrophages sensitizes chronic lymphocytic leukemia to apoptosis and inhibits disease progression. Cell Rep..

[31] Balachander G.M., Talukdar P.M., Debnath M., Rangarajan A., Chatterjee K. (2018). Inflammatory role of cancer-associated fibroblasts in invasive breast tumors revealed using a fibrous polymer scaffold. ACS Appl. Mater. Interfaces.

[32] Ouyang L., Highley C.B., Sun W., Burdick J.A. (2017). A generalizable strategy for the 3D bioprinting of hydrogels from nonviscous photo-crosslinkable inks. Adv. Mater..

[33] Gomez-Roman N., Stevenson K., Gilmour L., Hamilton G., Chalmers A.J. (2017). A novel 3D human glioblastoma cell culture system for modeling drug and radiation responses. Neuro. Oncol..

[34] Ma L., Barker J., Zhou C., Li W., Zhang J., Lin B. (2012). Towards personalized medicine with a three-dimensional micro-scale perfusion-based two-chamber tissue model system. Biomaterials.

[35] Emami A., Shojaei S., Da Silva Rosa S.C., Aghaei M., Samiei E., Vosoughi A.R. (2019). Mechanisms of simvastatin myotoxicity: the role of autophagy flux inhibition. Eur. J. Pharmacol..

[36] Sanchez-Cortes J., Mrksich M. (2009). The platelet integrin alphaIIbbeta3 binds to the RGD and AGD motifs in fibrinogen. Chem. Biol..

[37] Krawczyk M.A., Styczewska M., Sokolewicz E.M., Kunc M., Gabrych A., Fatyga A. (2019). Tumour expressions of hypoxic markers predict the response to neo-adjuvant chemotherapy in children with inoperable Rhabdomyosarcoma. Biomarkers.

[38] Dastghaib S., Shojaei S., Mostafavi-Pour Z., Sharma P., Patterson J.B., Samali A. (2020). Simvastatin induces unfolded protein response and enhances temozolomide-induced cell death in glioblastoma cells. Cells.

[39] Samiei E., Seyfoori A., Toyota B., Ghavami S., Akbari M. (2020). Investigating programmed cell death and tumor invasion in a three-dimensional (3D) microfluidic model of glioblastoma. Int. J. Mol. Sci..

[40] Shojaei S., Koleini N., Samiei E., Aghaei M., Cole L.K., Alizadeh J. (2020). Simvastatin increases temozolomide-induced cell death by targeting the fusion of autophagosomes and lysosomes. FEBS J..

[41] Gkretsi V., Stylianou A., Louca M., Stylianopoulos T. (2017). Identification of RAS suppressor-1 (RSU-1) as a potential breast cancer metastasis biomarker using a three-dimensional in vitro approach. Oncotarget.

[42] Garreta E., Gonzalez F., Montserrat N. (2018). Studying kidney disease using tissue and genome engineering in human pluripotent stem cells. Nephron.

[43] Megiorni F. (2020). Epigenetics in Rhabdomyosarcoma: cues to new biomarkers and targeted therapies. EBioMedicine.

[44] McCarthy N., Dolgikh N., Logue S., Patterson J.B., Zeng Q., Gorman A.M. (2020). The IRE1 and PERK arms of the unfolded protein response promote survival of Rhabdomyosarcoma cells. Cancer Lett..

