# Supplementary figures and images for: A bioengineering method for modeling alveolar Rhabdomyosarcoma and assessing chemotherapy responses
Source: MethodsX. 2021 Jul 27;8:101473. doi: 10.1016/j.mex.2021.101473 (PMC8374652; doi:10.1016/j.mex.2021.101473)

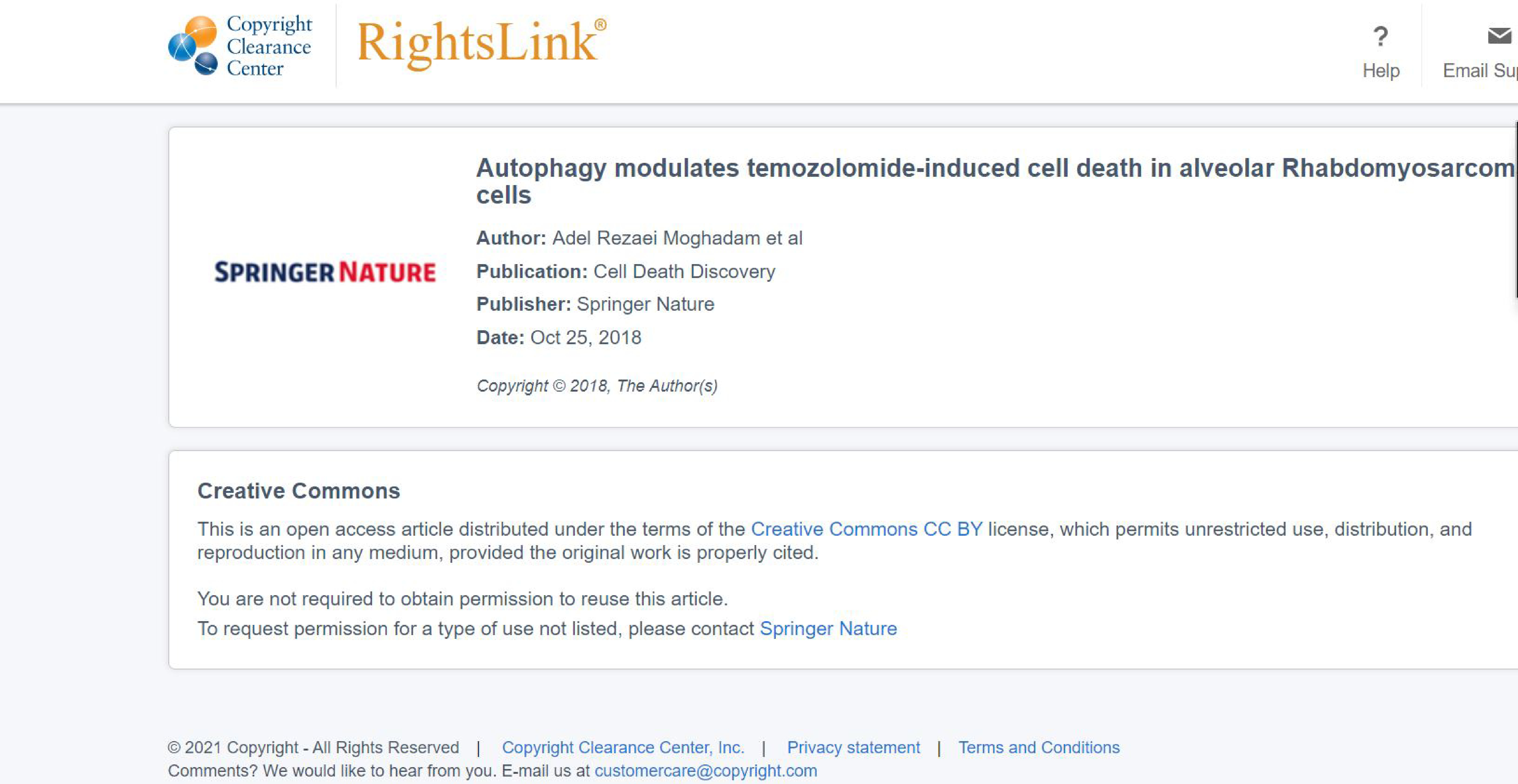

Supplement: Supplementary file 1 [file mmc1.jpg]
